# Medico-Chirurgical Transactions

**Published:** 1817-04

**Authors:** 


### PART II.

### COMPREHENSIVE ANALYTICAL REVIEW OF MEDICAL LITERATURE.

"Tros, tyriusve, nobis nullo discrimine agetur."

### *Medico-Chirurgical Transactions, Vol. VII. Part II.*

Concluded from page 224.

ART. 1. *On the Treatment of Sinous Ulcers.* By H. DEWAR, M. D. &c. &c.

THIS is a discourse in good set terms, and although somewhat prolix, is not devoid of interest or utility. The tediousness and want of success so often attending the treatment of sinous ulcers have occasioned much variety of opinion aud directions on this point of practice. We are most generally, however, advised to lay open a sinus along its whole course, for the complete discharge of the matter; or to make counter-openings for that purpose. Few mention the effects of compression, and none describe with fulness the mode of applying it. It is to extensive sinuses among the muscles of the thigh, where the benefits of compression are most conspicuous, that Dr. D. confines his attention in this paper. These occur chiefly in consequence of abscesses formed under the *fascia lata*, and of gun-shot and other deep-seated wounds. The tough unyielding substance of the fascia prevents the matter from pointing outwards, so that it extends interiorly in all directions, favoured by the looseness of the cellular membrane, the number and length of the muscles, their narrow and distant attachments, and divergency where connected with the bones of the pelvis. The methods of dressing in general use, may be reduced to two: One is, to put a loose roller over the whole; or to be satisfied with a covering in the form of a towel containing a large poultice, that the parts may be kept easy, and that the matter may have a free exit. "Under this management, there may be an overflow, but there is no evacuation." A laborious, and altogether ineffectual part of daily duty, is to force out the discharge by pressing the integuments from the extremities towards the orifice of the cavity. But the disease extends, and no effort is made to produce adhesion. The other mode of bandaging, is to apply a roller with moderate tightness, but equal pressure, over the whole thigh; beginning at the knee joint, and carrying it regularly upwards by successive turns, passing it over the orifice in its way, and fixing it at the upper part of the thigh. This application is equally ineffectual as the preceding. It cannot produce an immediate adhesion of parts so extensively divided, and kept asunder by a profusely secreted pus. In general it so presses the orifice itself as to retain forcibly the whole pus formed between the times of dressing, and thus to increase the irritation excited by the presence of a foreign body.

"The following is the method to be substituted for the preceding expedients. A few turns of the roller should first be made *with considerable pressure* over one extremity of the femur, and then over the other, so as to reach with all possible certainty the extremities of the large sinus; into which the whole cellular interstices of the parts have been converted. It is safer to begin beyond the sinus than to run any risk of falling short of its extremities; and, in some cases, it might be proper to increase our security by means of partial compresses extending somewhat higher than it is possible to apply the turns of the roller itself. It is now fixed in its situation with a pin. A considerable pressure is easily borne, as no high inflammation is present, and the evacuation of the pus, by reducing the circumference of the limb, soon relieves the veins from any turgescence arising from the pressure to which they may have been at first subjected. In country practice, when a Surgeon has been newly called to an old case of this kind, and a considerable interval may elapse before he is to repeat his visit, the swelling of the lower part of the limb may be obviated by bandaging it upwards from the toes. After fixing the bandage on the thigh at the degree of pressure which I have described, the Surgeon may, if he chooses, make two or three lighter turns on the tumid part, to assist the depletion of it; taking care that these press so lightly, as in no degree to counteract the operation of the first turns made at the extremities of the sinus. The change which this application produces is almost immediate. Part of the matter with which the integuments had been distended, is irresistibly forced a certain way towards the orifice; and no newly secreted matter is suffered to lodge in that quarter. On the second day the limb is found somewhat reduced in size, and the bandage may now be applied more extensively. On the third day it may be so applied as to be kept on for several days without alteration. The same degree of pressure is always to be continued over the extremities of the sinus, and several additional turns are to be made gradually looser, alternately above and below the orifice, and approaching to it in both directions, but not reaching it. If there are two orifices, one of them, by which the matter can be freely brought away, is to be left uncovered with the bandage, and the other allowed to heal up. There is no necessity for selecting the most dependent one for that purpose, as any advantage derived by the tendency given to the course of the matter by its own weight, is not worthy of attention under a treatment implying means of evacuation otherwise so powerful. The anterior orifice will often be found the most eligible, as it is examined and dressed with greatest convenience. During the alternate application of the bandage to the upper and lower part of the thigh, it is frequently and variously *crossed* on the side of the limb opposite to the open orifice, and thus a propulsion of the pus is commanded in every direction to that outlet. A considerable part of the surface surrounding it is left uncovered, and the bandage is finally fixed. Over the orifice such light dressings are subsequently applied as will make no resistance to the discharge of the purulent matter. The firm propelling bandage is kept on without alteration, except when it becomes loose in consequence of a reduction in the size of the limb; although cleanliness requires the dressing immediately over the orifice to be changed daily or oftener. Thus all unnecessary trouble is prevented, an object which is sometimes of importance in securing the more perfect performance of those offices which are really necessary." p. 489.

When the pus is the only foreign body contained in the cavity, the process of adhesion soon begins at the extremities of the sinus. The process of adhesion advances by successive steps towards the orifice, which, in the end, requires little pressure. The discharge quickly diminishes, and shews a proportional diminution of internal disease; and the parts heal in the same kindly manner as a superficial abscess among firm integuments. This mode of bandaging will possess considerable advantages in cases of more complicated disease. If pieces of cloth, splinters of bone, or other foreign bodies, are present, it will tend to expel them: and if not, yet, by reducing the local disease to the spot in which they are lodged, it procures for the practitioner sure information of their presence. When the bone is fractured by a musket shot, it will have the effect of reducing the disease more nearly to that of a common compound, and soon to that of a simple fracture. In the arm and leg, there will be no occasion to deviate from the exact form of the bandage already described. We think Dr. Dewar's hint is worthy of notice by our surgical brethren.

ART. 2. *Some Observations on a Species of Pulmonary Consumption very frequent in Great Britain.* By A. P.W. PHILIP, M. D. &c. of Worcester.

Dr. PHILIP's name is so well known and universally respected in the medical world, that any thing bearing his signature is entitled to attention. The subject is also extremely interesting to every inhabitant as well as every practitioner in this land of fogs and storms.

Dr. P. conceives that the species here described is, under the common treatment, nearly as fatal as the other species of the disease; but under that which is suited to it, its progress, especially in the early stage, may be arrested.

*Prominent Symptoms.* Spirits, from the beginning, more or less depressed; countenance unusually sallow; cough, at first dry, or only followed by a little mucus; comes on in violent fits, the intervals being nearly free from this symptom. The coughing fits generally succeed a meal, and on lying down: frequently on lying on the left side; less apt to come on when the patient lies in the recumbent position on the back, with the shoulders raised. In this, as in other species, the cough is troublesome after awaking in the morning. In progress, the cough becomes more frequent; less decidedly in fits; attended with more copious expectoration, at first limpid and glairy, by degrees mixed with a small portion of an opake, pus-like substance, the proportion of which gradually increases. Dr. P. has seen half a pint or more of this, mixed with tough phlegm, expectorated daily; the other symptoms being comparatively mild. Bloody expectoration is by no means uncommon in this species of phthisis. When mixed with a transparent fluid *only*, there may be good hopes of recovery. If there be no appearance of blood, there may be good hopes of recovery, if the disease have not lasted long. When the expectoration assumes a sanious appearance, it indicates nearly as much danger as in the other species. Dr. P. wisely waves all discussion respecting the means of distinguishing pus from mucus. The only criteria which he has found necessary in practice is the pus-like appearance, and its sinking in water, when so agitated as to separate it from the tough mucus. The breathing, in this species, is sometimes more oppressed by the recumbent posture than in other forms of the disease; and is more frequently attended with a sense of tightness across the pit of the stomach. There is very rarely, except in the advanced stages, that marked dyspnœa on exercise, which so frequently attends even the commencement of other forms of the disease.

There is often little or no pain. Sometimes a dull pain in the pit of the stomach, or pretty low down in the left side of the chest; more rarely in the right. Hardly ever a fixed pain high in the chest; sometimes an uneasy sensation, and a sense of oppression under the sternum; sometimes darting pains in various parts of the chest; frequently in more distant parts, particularly the limbs, back and shoulders, head, &c. The hectic fever is hardly ever so completely formed in the earlier stages, as in other species of phthisis: sometimes a copious purulent expectoration with but slight fever, and that not of the hectic kind, the skin remaining dry in the morning, with little or no evening exacerbation. Emaciation seldom so rapid as in other species, but is *pari passu* with the fever. Such is the *modification* of symptoms in this species; but a diagnosis resting on *modification* alone, must always be fallacious. Fortunately there are here superadded to the usual symptoms of phthisis, others which are peculiar to this species, viz. *derangement of the digestive organs*. Flatulence; irregularity of bowels; furred tongue; impaired appetite; discoloured fæces; tenderness and fulness of the epigastric region are almost always present, the pulmonary symptoms keeping a proportionate pace with them. In advanced stages, the symptoms of this species amalgamate with those of common phthisis, while the fulness and tenderness of the epigastric region are often lessened, and sometimes totally disappear—a bad prognostic. The patient sinks with the usual phthisical appearances; but if the affection of the digestive organs have been neglected, ascites is frequently an attendant here; a symptom which rarely supervenes on other cases of phthisis.

*Causes.* These are as numerous as those of other forms of the disease. This species indeed is not induced by the inhalation of dust; other diseases of the lungs; unequal pressure of bones, &c. &c. But then those causes which derange the function and structure of the chylopioetic organs, operate here. Hence it may be termed, D. P. thinks, "*Dyspeptic phthisis*"; we would rather term it, "PHTHISIS GASTRO-HEPATICA."

*Post mortem appearances.* The lungs present the same appearances as in other species, "*but we almost always find at the same time, either a diseased state of the liver, or traces of disease having existed there.*" Dr. P. has repeatedly found those parts of the lungs in the neigbourhood of the liver, alone affected, the left side appearing sound, or nearly so. The hepatic affection, however, seems to have little immediate share in the cause of death, the patient living till almost the whole of the lungs are rendered incapable of their function.

Disease of the spleen is also a common attendant on gastro-hepatic phthisis.

*Nature of dyspeptic Phthisis.* A question of the first importance in the treatment of this disease here arises. What is the nature of the sympathetic relation between the lungs and digestive organs in this complaint? Is the one a consequence of the other? Or, are they *simultaneous effects of one common cause?* Dr. P. thinks not: because in by far the majority of cases, he found the gastro-hepatic precede the pulmonic affection. A diseased state of either set of organs may produce that of the other;

"But the tendency of disease to spread from the digestive organs to the lungs is much greater than *vice versa.* When we find also that every thing which relieves the gastro-hepatic malady, relieves at the snme time the pulmonic, the inference appears unavoidable that the pulmonary disease arises from that of the digestive organs."

Dr. P. well observes, that the effect of the sympathy which exists between different parts of the animal body in the production of disease, does not seem to have attained the attention it deserves.

"It is well known that nervous affections will, if I may use the expression, mimic the symptoms of almost every disease; but it does not seem to be generally admitted, that if this mimic disease is kept up for a certain length of time, it will be converted into the real disease."

Thus disordered digestion produces palpitation and syncope; and if these symptoms continue to recur for a great length of time, they end in real disease of the heart. The same cause also produces, first, drowsiness and inability; but ultimately real organic disease of the brain; first cough and dyspnœa, ultimately real disease of the lungs. But although no real disease at first exists in the organs sympathetically affected, yet we may fairly conclude, that some degree of debility, aptitude, or predisposition prevailed there, giving, as it were, a direction to the sympathetic influence.

For the *modus agendi* of this sympathy, indeed, we cannot account; but it teaches us an important lesson in the prevention of disease, by watching the first beginnings of sympathetic affections with care. It teaches us a lesson of equal importance in the treatment of diseases; to make ourselves minutely acquainted with their history, in order to ascertain, if possible, which was the primary, which the secondary seat of the malady.

*Treatment of dyspeptic phthisis.* In dyspeptic phthisis we always perceive, sooner or later, some fulness and tenderness of the epigastric region. It is *after* these have supervened, that the chylopoietic derangement affects the pulmonary function; and it is in proportion as we relieve the *former*, that we assuage the latter. The fulness and tenderness depend on affection of the liver, and *their* degree is a pretty correct measure of *its* degree. This species of phthisis may be divided into four stages, in which the prognosis and mode of treatment are different. 1mo. The pulmonic affection is merely sympathetic, and ceases with the removal of its cause. This stage is short in duration; mild in symptoms; (particularly the fever) accompanied by no expectoration except some phlegm with the cough. 2do. The sympathetic has produced actual disease in the lungs, indicated by some degree of inflammation in the bronchia, and admixture of pus-like substance in the expectoration, sometimes blood. The tendency to fever is now greater, yet seldom in the hectic form. It is at this period that tubercles seem to form. 3tio. *Omnia exacerbata.* The fever becomes completely hectic; the expectoration purulent, or mixed with blood, and assuming all the various appearances observed in the last stages of other phthises. 1mo. In the first stage the disease generally yields readily, unless the dyspeptic symptoms are peculiarly obstinate, or there is a strong disposition to a tubercular state of the lungs; a circumstance which may baffle the best concerted treatment. Scrofulous enlargement of the more external glands is an unfavourable symptom; yet suppuration of these may probably often ward off disease in the lungs. This stage then being free from the above, yields to the usual means of relieving the cough and tendency to fever, combined with light diet, open bowels, and occasional doses of blue pill or calomel, to preserve a sufficiently copious and healthy secretion of bile. To these means, stomachic medicines, especially when the appetite is impaired, may be superadded. Dr. P. has found Extr. chamemeli combined with a small quantity of ol. carui the best stomachic.

2do. The second stage requires a different treatment. Dr. P. while he attended to the state of the bowels, has been in the habit of giving in this stage, one grain of the blue pill combined with some mild stomachic, two or three times in the twenty-four hours, continuing it either till the epigastric tenderness yielded, or the gums appeared redder and fuller than usual. As the epigastric tenderness abates, and the fæces assume the natural appearance, the pulmonary symptoms will, in a majority of cases, gradually give way. Dr. P. has found these small doses of the mercurial succeed better than larger ones.

*Local Means.* A succession of small blisters, sometimes preceded by the abstraction of a few ounces of blood from the epigastrium. Where the disease is obstinate, a permanent discharge by means of a seton. For promoting a regular and healthy secretion of bile, small doses of Epsom salts are useful auxiliaries to the mercurial. Dr. P. speaks highly of the dandelion, taken either in substance, decoction, or the fresh expressed juice, in doses of two or three table spoonsfull thrice a day in camomile tea. With this last medicine, half a grain of blue pill thrice a day will be sufficient.

If neither the tenderness of the epigastrium be removed, nor the gums affected by these means in a fortnight, the quantity of blue pill is to be increased till one of these effects results. If either take place without relieving the pulmonary symptoms, the prognosis is bad. If the epigastric tenderness continue, the hepatic affection is unusually obstinate. If this be removed without relief of the pulmonary symptoms, then the lungs are most probably too far diseased. "It is surprising from what states the lungs will sometimes recover, when relieved from the irritation of the hepatic affection." When the failure of relief proceeds from obstinacy of the hepatic affection, some hope arises from a fuller mercurial course; but this hope is often fallacious, since the means that relieve the hepatic will too often be found to increase the pulmonic lesion. It is the *anceps remedium,* however, and the only one.

It sometimes happens that the tenderness of the epigastrium is wholly, but the pulmonary symptoms only partially, relieved by the above plan. In this case, the hepatic affection is apt to recur, always bringing with it an increase of the pulmonary symptoms, till the structure of the lungs is at length destroyed. If the hepatic affection be neglected, the fatal termination will be rapid; if carefully watched and relieved, as it appears, the case is protracted, and the decline of the patient is gradual. Under any plan, these cases generally prove fatal at last; and the only chance of success is by constant attention to the hepatic affection. The most fatal case is where the seat of the disease is wholly transferred from the liver to the lungs, as happens frequently in the last stage of this species. In this case, there is no hope; whereas, while the hepatic affection continues to recur, there is always some chance of success. With respect to the treatment of phthisis generally, Dr. P. has little to offer. He thinks he has found a combination of the extracts of white poppy and conium the best anodyne in this form of the disease. Opium constipates, and hyosciamus is uncertain. When the epigastrium is very tender, animal food and fermented liquors are peculiarly injurious. Mercury he has never found useful in the common forms of phthisis. "An alterative plan, conducted in the way above pointed out, I have found equally serviceable in what are called nervous diseases, when they arise, as frequently happens, from the above state of the digestive organs. Thus habitual depression of spirits, head-ache, palpitation, and many other complaints of the same description, may be relieved." *535.*

We have dwelt long on this article, because we greatly respect every thing that comes from the pen of Doctor Philip; and because, in reality, it is by far the most interesting article in the whole volume.

Art. 3. *Facts illustrating the Effects of the Venereal Disease on the Fœtus in Utero, and the Modes of its Communication.* By William Hey, Esq. of Leeds.

From a gentleman fifty-seven years engaged in the practice of Midwifery, any article of this kind must be interesting; but from Mr. Hey it is doubly so, as he is a man of no ordinary stamp.

Mr. H. says, that he cannot better describe the disease in question than by relating the last case which has occurred in his practice.—A poor woman brought to his Surgery an infant betwixt two and three months old. It had been extremely fretful, without disordered bowels; its voice had grown stridulous; it had upon its chin a scaly eruption, extending to the angles of the lips, and its body was covered with copper-coloured spots. The mother was suspected to have had the venereal disease.—He directed half a grain of the sub. hyd. with a few grs. of the pulv. tragac. comp. to be given to the child twice a day, and to continue it seven or eight days. At the end of this period the infant was much improved in health; its fretfulness had ceased; voice scarcely stridulous; copper-coloured blotches beginning to fade; eruption on the chin diminished. Perseverance was advised. Here Mr. Hey remarks, that infants bear the use of mercurials in doses that will affect adults, without any apparent disturbance of the animal functions. He generally gives the foregoing dose to children within the year, without producing any salivation, which is indeed seldom if ever excited in infants. When the bowels are relaxed, he joins with it a quarter or half a minim of tinct. opii, or gives the hydrarg. c. creta.

This disease is often, in infants, accompanied by extensive desquammation of the cuticle. Several instances occurred in Mr. Hey's practice, where the mother, having once been affected with the disease, has communicated it to two, three, or four children in succession, without any fresh infection. In 1770, a blind woman, who gained her bread by drawing the breasts of women, became affected with ulcers at the angles of her lips, which were judged to be venereal. They did not heal till she underwent a course of mercury. Several women, whose breasts she drew, became infected, as in the following instance.

Mrs. B. in three or four weeks after her breasts were drawn, perceived a swelling in the axillary glands, and complained of soreness in her throat. A judicious Surgeon pronounced the case venereal, and treated it as such. During the treatment she became pregnant, but persevered till the fifth month. Mr. H. attended her in labour, and saw nothing amiss in the parts of generation; and she asserted that she never had any complaint there. She became pregnant again in 1772, and was delivered in February 1773, of an apparently healthy child, which she herself suckled. In six weeks a syphilitic eruption appeared on the legs and arms of the child. Mr. H. immediately put parent and infant on a proper course, and the child was soon freed from the eruption. In October following, two or three small ulcers appeared on the labia pudendi of the child, and the mercurial course was resumed. The ulcers soon healed. In May 1774, the nostrils became sore, and the integuments of the nose were also tender. The child's voice grew hoarse. The mercurial course was resumed, and continued two months. After this there was no recurrence of the disease. In June 1775, Mrs. B. bore another child, apparently healthy at birth, but in a few weeks copper-coloured blotches appeared on the skin, and were removed by a mercurial course. After some time the blotches re-appeared, accompanied by a small ulcer on the labium pudendi; the child was completely cured by a repetition of the course.

We think the above were *syphiloid* rather than syphilitic diseases. Mr. Hey believes, "that a man may communicate the lues venerea after all the symptoms of the disease have been removed, and he is judged to be in perfect health." We doubt both this and the following case. A gentleman consulted Mr. Hey, while tears flowed from his eyes. He had married a lady whom he tenderly loved, but feared he had injured her. He had had the disease before marriage, but believed himself perfectly cured, and remained free from complaint to the present moment. Mr. Hey visited the lady; found the labia pudendi beset with irregular fissures and condylomata. A discharge of puriform matter also issued from the vagina. She was in the seventh month of pregnancy. A *gentle* mercurial course was pursued; and before parturition the diseased parts were healed. The child had an universal desquammation of the cuticle. It continued well for a month, then grew fretful, but without indication of disorder in the primæ viæ; its voice got hoarse; and a number of copper-coloured spots came out upon the skin. A scaly eruption also appeared on the chin, and the anus also shewed an unnatural redness. A mercurial course removed all these. A relapse took place, but the same treatment removed the complaint. Mr. H. offers the following considerations to those who doubt the propriety of the name given to the foregoing diseases.

When Mr. Hunter first brought into notice syphiloid complaints, he rested the distinction on the fact that pseudo-syphilis was cured *without* mercury, and therefore it was proved to be of a different nature from real syphilis. "Now, (says Mr. Hey) if this reasoning be allowed to have validity, we may suppose the converse of it also to be valid. If the disease in question have the usual symptoms of syphilis, *and will yield to no other remedy than mercury*, we may fairly conclude that it is syphilitic." In the first place, it does not appear that Mr. Hey tried any other remedy than mercury; and consequently, one part of the position is defective. In the second place, we know from abundant experience, that decidedly *syphiloid* diseases will yield under a *gentle* course of mercury; which, with decoction of sarsaparilla and attention to the bowels and biliary secretion, will be found the best means of removing them. If the lady's case was *real* syphilis, how did the gentleman escape re-infection? We believe the question will puzzle Mr. Hey. In short, we have no doubt of the whole of the preceding diseases being pseudo-syphilitic.

ART. 4. *On the Medicinal Properties of Stramonium;* By Dr. MARCET.

THIS is a lengthy paper, [30 pages] but will not detain us very long. The preparation which Dr. Marcet uses is the extract of the seeds, and also of the plant, made by Mr. Hudson, chemist and druggist, in the Haymarket.—Dr. M. gives the preference to the extract from the seeds.

Without pretending to have yet acquired a competent knowledge of this medicine, Dr. Marcet may at least state that the most common effect of stramonium when administered in appropriate doses, from an eighth of a grain to a grain, in chronic disease, attended with acute pain, is, to lessen powerfully, and almost immediately, sensibility and pain; to occasion a sort of nervous shock, which is frequently attended with a momentary affection of the head and eyes; with a degree of nausea; and with phænomena resembling those that are produced by intoxication; to excite in many instances nervous sensations, which are referred to the œsophagus, or bronchiæ, or fauces, and which sometimes amount to a sense of suffocation; to have rather a relaxing than an astringent effect; to have no marked influence on the pulse; to produce but a transitory and inconsiderable dilatation of the pupil; to have but little immediate tendency to produce sleep, except from the comparative ease and serenity which generally follow the symptoms above described. In some instances the beneficial effects are obtained without the patient experiencing any of the uneasy sensations above mentioned, while in a few others, the unpleasant consequences take place without any subsequent benefit. We shall now condense a few of the cases by way of illustration.

CASE I. J. E. ætat. 30, admitted April 5, 1816. Symptoms of veteran sciatica with suspicion of diseased hip joint. Pains in the hip and knee excruciating. Cicuta, hyoscyamus, opium, blisters, were tried in succession, without any relief. 13th April, administered half a grain of extract of stramonium thrice a day. The relief was immediate, and very striking, but she uniformly complained of a peculiar sensation of heat in her throat, with a spasmodic affection in her breathing, after each dose. These disappeared in a few minutes. On the 5th of June, the pains were so much subdued, that she was discharged cured.

Case 3. Mrs. T. ætat. 48, had a scirrhous tumour in the right breast for many years. Mr. John Pearson tried the effects of pressure with some advantage, in respect to the size and surface of the tumour. Whilst under this treatment, however, she was suddenly seized with acute pains in her loins; and, three or four weeks afterwards, when apparently mending, she was suddenly affected with what she called a crawling sensation in her left hip and thigh, extending down to the knee and leg, and occasionally shooting towards the groin and pubes. This sensation was almost immediately converted into pain of the most acute and excruciating kind, especially on attempting to move the affected parts, but without fever. Bleeding, blistering, opiates, cicuta, hyoscyamus, were tried in vain. Opium gave temporary relief, but induced an obstinate constipation. The pressure on the breast was now discontinued, in consequence of the exquisite pain it produced in the loins and thigh. These symptoms had continued two months, when Dr. Marcet and Mr. Pearson prescribed a quarter of a grain of the extract from the seeds thrice a day, which was increased the following day to three-eights of a grain. The result, even from the first dose, was very remarkable. In a few minutes, a peculiar and indescribable sensation was felt in the throat, which she compared to a current of wind rushing up and down the throat, and producing a slight sense of suffocation. In fifteen minutes the *pain* subsided sensibly, and soon afterwards entirely, leaving an uncommon feeling of serenity and repose, unaccompanied by drowsiness. These effects were reproduced whenever she took the pill; but after a few doses, the recurrence of pain altogether ceased, and the stramonium was soon laid aside. She was now seized with inability to expel urine, and want of inclination to it. The catheter drew it off.

Next day, the bowels were quite inert; and castor oil procured stools, but with little or no control over the sphincters. From this time no urine was passed but *via* the catheter; and no stools procured but by cathartics. Being refreshed, however, by the absence of pain, her strength seemed to recruit, and she recovered some degree of appetite and cheerfulness; but she never ventured to sit up in bed, from a sense of weakness in the loins, and a dread of pain. The operation of strapping the breast was resumed, but soon discontinued on account of the fatigue it occasioned. The further details of this interesting and melancholy case are not given; but Dr. M. says,

"It will suffice to add, that the paralytic symptoms did not in any degree abate, and that the strength continued gradually to decline, though the pain did not return."

After a long confinement, she was carried in a litter into the country, the change affording her exquisite enjoyment. Soon after this, however, some gangrenous spots appeared on the sacrum, and became highly inflamed and painful. The strength and digestion now failed, and she sunk into the arms of Death.

Dr. M. informs us, that there were various conjectures respecting this case. We have little difficulty in believing that the original complaint in the breast was a *constitutional* disorder, and not to be removed by *local* means, except at the expence of the general health, or a conversion of the disease into another and more dangerous form. We believe that the day is not far distant, when these things will be more known and better understood than at present.

Case 4. *Sarah Mears, ætat.* 23. This is an extraordinary case, which has excited much curiosity lately in Guy's Hospital. The original symptoms were, five or six years ago, a tumour, first inclining to the left side, but afterwards occupying the whole of the abdomen, occasioning in its progress exquisite pain, with fever and extreme irritation, but without emaciation, or permanent injury to the constitution or functions of the abdominal viscera. This tumour gradually increased to an enormous size, so as greatly to exceed that of a woman in the last stage of pregnancy. The pain became more and more intense, till at last enormous quantities of a sanious or puriform fluid, mixed with blood and serum, were simultaneously discharged, partly by vomiting, and partly by the vagina and the rectum, and the patient soon recovered. In the course of a few months, however, the complaint gradually returned with similar symptoms, which were again relieved in the same manner; and the tumour has now, for the eleventh time, gone through the process of filling and bursting, with extreme pain, and subsequent sudden relief, in the way above described. It was on this last occasion, during the formation of the tumour, the pain being at the highest pitch, and opium affording but little relief, even in doses of eight or ten grains, that the stramonium was tried in the dose of only half a grain three times a day. This remedy uniformly produced giddiness and dimness of sight, but the pain was immediately relieved for a few hours. After continuing the stramonium five days, the tumour burst through the usual outlets, and there being a truce to the pain as formerly, the medicine was discontinued. The cyst is not now allowed to fill; on the contrary, the moment the throbbing and fulness recur, the accumulating fluid is forced out by external pressure, and discharged by the rectum and vagina. This operation is repeated once or twice a week, and the reproduction of the tumour, to any extent, is thus prevented. Such is the tendency to inflammation in the diseased part, that cupping and bleeding are constantly necessary, and have been practised more than two hundred times during the period of her illness, and are still required. Latterly, the urinary organs are much affected, and the use of the catheter is daily necessary. An habitual state of pain and irritation now renders narcotics necessary, and stramonium affords the greatest relief.

This remedy certainly deserves a fair trial, and we ourselves are giving it one; the results of which we shall make known.

Upon the whole, the 7th vol. of these Transactions is not, we think, inferior to the majority of its predecessors. The work promises to be one of national importance and general utility, honourable to the upper and valuable to the lower orders of the profession.

